# Prevalence of behavioral risk factors of cardiovascular diseases and associated socio-economic factors among pregnant women in a rural area in Southern Nepal

**DOI:** 10.1186/s12884-018-2122-5

**Published:** 2018-12-07

**Authors:** Rajan Paudel, Kwan Lee, Jitendra Kumar Singh, Seok-Ju Yoo, Dilaram Acharya, Rajendra Kadel, Samaj Adhikari, Mohan Paudel, Narayan Mahotra

**Affiliations:** 1 Department of Community Medicine and Public Health, Maharajgunj Medical Campus, Institute of Medicine, Tribhuvan University, Kathmandu, Nepal; 2 Department of Preventive Medicine, College of Medicine, Dongguk University, 123 Dongdae-ro, Gyeongju-si, 38066 Republic of Korea; 3 Department of Community Medicine and Public Health, Janaki Medical College, Tribhuvan University, Janakpur, Nepal; 4 Personal Social Services Research Unit, London School of Economics and Political Science, London, UK; 5 Maharajgunj Medical Campus, Institute of Medicine, Tribhuvan University, Kathmandu, Nepal; 6 Southgate Institute for Health, Society and Equity, Flinders University, Adelaide, Australia; 7 Department of Physiology, Maharajgunj Medical Campus, Institute of Medicine, Tribhuvan University, Kathmandu, Nepal; 8 Department of Community Medicine, Kathmandu University, Devdaha Medical College and Research Institute, Rupandehi, Nepal

**Keywords:** Behavioral risk factors, Cardiovascular disease, Pregnant women, Socio-economic predictors, Nepal

## Abstract

**Background:**

Cardiovascular diseases (CVDs) have dramatically infiltrated populations living in abject poverty in Low- and Middle-income Countries (LMICs), and poor maternal and child health outcomes have been frequently reported for those with CVD risk factors. However, few studies have explored the behavioral risk factors of CVDs among pregnant women in rural settings. This study aimed at determining the prevalence and identifying the socio-economic predictors of behavioral risk factors of CVDs among pregnant women in rural area in Southern Nepal.

**Methods:**

A Community-based cross-sectional study was conducted in 52 clusters of Dhanusha District of Nepal in a total of 426 pregnant women in their second trimester using multistage cluster sampling method. Multivariable logistic regression model was used to assess independent associations between behavioral risk factors during pregnancy and maternal socio-economic characteristics.

**Results:**

Of the 426 study participants, 86.9, 53.9, 21.3 and 13.3%, respectively, reported insufficient fruits and vegetables consumption, insufficient physical activity, tobacco use, and harmful alcohol drinking. Socio-economic factors significantly associated with more than one behavioral risk factors in expectant mothers with a primary level education (adjusted odds ratio (AOR) 2.78; 95% Confidence Interval (CI) (1.35–5.72)), 20–34 years age group (Adjusted Odds Ratio (AOR) 0.27; 95% CI (0.13–0.56)), and those with the highest wealth index (AOR 0.36; 95% CI (0.16–0.84)).

**Conclusion:**

Higher prevalence of behavioral risk factors for CVDs and their socio-economic factors prevailing among pregnant women living in rural Nepal call for immediate health promotion interventions such as community awareness and appropriate antenatal counseling.

## Introduction

Cardiovascular diseases (CVDs) include disorders of heart and blood vessels, and are usually associated with atherosclerosis, heart attacks, strokes, valvular diseases, congenital heart diseases and arrhythmia [1]. Mortality resulting from CVDs reached 17.7 million globally in 2015, which represented 31% of all global deaths. Of the CVD deaths, more than three quarters took place in low- and middle-income countries (LMICs) [2]. Cardiovascular diseases, which are typically viewed as diseases of the wealthy, have dramatically infiltrated those living in abject poverty in LMICs [3], and accounted for a substantial burden of non-communicable diseases in Nepal. Studies [4, 5] revealed a prevalence of about 5% for coronary heart diseases among adult population in Nepal. A study by Dhungana et al. found high prevalence of risk factors of CVDs such as smoking, alcohol consumption, insufficient fruit and vegetable intake, daily salt intake, overweight and obesity, and hypertension among rural Nepalese residents [6].

Smoking, alcohol consumption, inadequate physical activity and inadequate fruit and vegetable intake are well-established modifiable behavioral risk factors of CVDs [7]. Globally smoking accounts for 10% of deaths attributed to CVDs, and this figure is gradually increasing in LMICs, alcohol is responsible for 6 and 1% of deaths among men and women, respectively, whereas inadequate physical activity and insufficient fruit and vegetable intake contribute nearly 33 and 10%, respectively, to the burden of ischemic heart disease [7].

The spectrum of CVDs in pregnancy encompasses congenital heart disease, valvular heart disease, ischemic heart disease, peripartum cardiomyopathy, hypertensive disorders, and venous thromboembolism. Furthermore, the global burden of CVDs in pregnancy has increased alarmingly in parallel with the rising prevalence of behavioral risk factors among women of reproductive age [8].

Addressing the risk factors of CVDs in pregnant mothers is crucial not just for reducing adverse pregnancy outcomes such as low birth weight, fetal and infant mortalities, and potential congenital defects, but for reduction of CVDs among mothers as well [8–11]. Cardiovascular diseases are exacerbated by physiological changes in pregnancy and contribute to maternal mortality and morbidity, and delay in health care response [12, 13], which has important impacts in resource constrained settings as rural Nepal. In Nepal, the 2013 non-communicable disease risk factor survey (STEPS) revealed national prevalence of tobacco use of 30.8%, alcohol consumption of 17.4%, inadequate fruit and vegetable consumption of 98.9%, and inadequate physical activity of 3.5% [14]. Studies on impact of the combined effects of behavioral health risk factors on longevity showed that these risk factors increased mortality rates in both genders [15, 16]. In addition, a higher percentage (9 to 15%) of pregnant women have been reported to drink alcohol during pregnancy in Nepal [17, 18]. However, these studies were limited in terms of describing the prevalence of behavioral risk factors and their predictors.

Identification of the behavioral risk factors of CVDs among pregnant women is an important global public health issue, but very limited studies have sought to identify behavioral risk factors of CVDs in pregnant women residing in rural settings. In this study, we aimed to determine the prevalence and to identify socio-economic predictors of behavioral risk factors of CVDs among pregnant women living in rural southern Terai of Nepal.

## Methods

### Study design, setting and sampling

We employed community-based cross-sectional study design. We used baseline data obtained from the ‘MATRI-SUMAN’, a randomized controlled trial of a capacity building and text messaging intervention designed to enhance maternal and child health service utilization among pregnant women residing in rural Nepal [19]. The baseline data included socio-demographic information and CVD risk factors - tobacco use, alcohol consumption, fruits and vegetables intake and physical activities.

The study was conducted in Dhanusha district of Nepal. Dhanusha, one of the 75 districts of Nepal, is situated in the southern part of the country (also known as ‘Terai’ in local language). Dhanusha is a district of considerable cultural and historic importance. It occupies an area of 1180 sq. km in southern Terai province and is located at an altitude of between 61 and 610 m. It borders three districts – Siraha, Mahottari and Sindhuli to the east, west, and north, respectively, and India to the south. The main residents of Terai are from the Maithili ethnic group and the adult literacy rate is 69% [20]. Administratively, this district comprises one municipality and 101 Village Development Committees (VDCs) and has an estimated population of 754,777 in 2011. VDC is the smallest administrative unit of a district [20, 21]. Data for this study were collected from eligible pregnant women from July to September 2015.

We used multi-stage cluster sampling technique to identify study participants. One primary health care center and one health post of Dhanusha district were selected purposively for the study. These health facilities were chosen because these were the only government-funded maternal and child health (MCH) services facilities available in these areas. No healthcare services were available from the private or not-for-profit sectors, which helped to evaluate the true impact of the government MCH services. These two health facilities consisted of 12 VDCs (54 wards), and populations in these VDCs ranged from 3500 to 19,000 [22, 23]. From each health facility, three VDCs were randomly selected so that study area constituted a total of six VDCs. In total, there were 54 clusters (nine in each VDC), but we excluded two clusters because they were situated in semi-urban areas. Complete enumeration of all households having pregnant women with gestational period between 13 to 28 weeks (2nd trimester) from the selected clusters of six VDCs was performed. Pregnant women were identified using antenatal care register maintained by Female Community Health Volunteers (FCHVs). Of the 453 eligible pregnant women, 426 gave consent to participate in the study. The details of study design can be found elsewhere [19].

### Data collection

Structured interview questionnaire was used to collect information from a pregnant woman and the interview was taken place at participant’s home. Enumerators were trained in research tools, interview process and measurement of variables. Field coordinators experienced in CVD risk factors supervised enumerators to ensure the quality of information. Preliminary questionnaires were checked and pre-tested in an adjacent district and necessary modifications were made. The questionnaire consisted of two parts: i) baseline characteristics of respondents; and ii) behavioral risk factors of cardiovascular diseases (tobacco use, alcohol consumption, fruit and vegetable consumption and physical activity).

The major outcome variables were behavioral risk factors of CVDs among pregnant women. CVDs risk factors were operationalized based on the standard definitions to ensure their comparability and to minimize error. If the participants had been using tobacco of any form (smoke or smokeless) and alcohol for last 30 days, they were considered as current users. Women who were using tobacco daily were defined as current daily tobacco users. Likewise, women having five or more drinks a day in past 30 days were labeled as current episodic heavy drinker. Consuming 341 ml of beer or 43 ml of home-made alcohol was equivalent to one standard drink (13.6 g of pure alcohol) [24]. If a person took at least five portions (400 g) of fruits and vegetables per day, it was considered as sufficient. One cup (250 ml) of raw green leafy vegetables and a half cup (125 ml) of cooked or chopped raw vegetables was equivalent to one serving of vegetables. Similarly, one portion of fruits was defined as one whole medium sized fruits like apple, banana or orange and ½ cup of chopped, cooked, canned fruit or a half cup of fruit juice [25]. We used “show cards” associated with food frequency questionnaire to collect data regarding fruits and vegetables intake during pregnancy.

We adopted World Health Organization (WHO) global recommendations on physical activity for health (adult) guideline to measure physical activity level among pregnant women in our study. Physical activity includes activities during travel, recreation and work. It was evident from this guideline that more active people are less likely to have metabolic and cardiovascular risks. WHO recommended moderate-intensity physical activity (at least 150 minutes per week) can help to reduce such risks among adults [26]. In rural (Terai) Nepal, women are generally engaged in the household chores and farm activities, and these activities fairly meet the criteria of moderate-intensity physical activity level [14]. Participants were asked various questions regarding their physical activity levels and based on their response the enumerator recorded hours spent on daily household, travel, recreational and occupational activities. Based on recorded hours of time on various activities, the researchers then recoded these activities into two equalized categories (i) less than 150 minutes per week of moderate-intensity physical activity (Physical inactivity) and (ii) more than 150 minutes per week moderate-intensity physical activity (Active physical activity).

The independent variables comprised: age, caste/ethnicity, place of origin, religion, education, occupation, wealth index, and parity, which were adapted from the Nepal demographic and population health survey 2011 [27]. Caste/ethnicity was based on the caste base system in Nepal: disadvantaged-Dalit, disadvantaged-Adibasi/Janajati, and advantaged-Brahmin and Chhetri [28].

### Data analysis

Data were compiled, checked, cleaned and verified carefully to ensure consistency and then entered into database systems using Epi-info data version 3.1. Data analysis was carried out using SPSS version 20 for Windows. Univariate analysis was performed to describe the distributions of the four risk factors of cardiovascular diseases (tobacco use, alcohol consumption, physical inactivity and insufficient fruit and vegetable consumption). Associations between various factors such as, ethnicity, women education and wealth index and CVDs risk factors were analyzed first by bivariate analyses using chi-square test. A multivariate regression model was used to assess independent associations between risk factors during pregnancy and maternal socio-economic characteristics. Factors which were significant (*p* < 0.05) in bivariate analysis were selected for multivariable analysis and adjusted odds ratios (OR) and 95% confidence intervals (95% CI) were obtained. A *p*-value of < 0.05 was considered statistically significant. Further, to determine the simultaneous presence of behavioral risk factors and possible predictors, a number of risk factors in each pregnant woman was recorded (range of 0–4 behavioral risk factors). A model was used for a number of simultaneous risk factors; each woman was dichotomized as having less than two or having two or more behavioral risk factors (< 2 vs. ≥ 2). This dichotomized variable was used as outcome variable [29].

### Ethical considerations

Ethical approval for Matri-Suman trial was obtained from the Nepal Health Research Council, Nepal (Approval no: 101), the ethics committee of the Institute of Medical Sciences, Banaras Hindu University, India (approval no: ECR/526/Inst/UP/2014 Dt.31.1.14), and from the District Public Health Office, Dhanusha, Nepal (Ref. 2245). The objectives of the study were clearly explained to all participants and written informed consent was obtained before data collection. Personal identifiers were removed before data analysis.

## Results

Table 1 shows the prevalence of behavioral risk factors for cardiovascular diseases. Of the 426 study subjects, majority (86.9%) of them reported insufficient fruits and vegetables consumption, more than half (53.9%) mentioned insufficient physical activity, 21.3% reported tobacco use of any form (smoke or smokeless), and 13.3% as having harmful alcohol consumption.

**Table 1:** Behavioral Risk factors of the study subjects

Behavioral risk factors	Yes	No	Total
	*n* (%)	*n* (%)	*N* (%)
Use of Tobacco (any form)	91 (21.4)	335 (78.6)	426 (100)
Use of alcohol	57 (13.4)	369 (86.6)	426 (100)
Insufficient fruit & vegetable intake	370 (86.9)	56 (13.1)	426 (100)
Insufficient physical activities	230 (53.9)	196 (46.1)	426 (100)

Table 2 presents the socio-demographic details and the prevalence of behavioral risk factors for cardiovascular diseases among expectant mothers. The highest percentage of the study subjects (68.1%) were from 20 to 34 years of age. Most study subjects were from upper caste (62.0%), of Terai origin (71.8%), and Hindus (91.8%). Slightly more than a quarter of the study subjects had never been to school (25.4%), 68.5% were engaged in occupation other than agriculture, 29.1% belonging to lower socioeconomic strata, and 39.2% were primipara (pregnant for the first time).

**Table 2:** Behavioral Risk factors associated with baseline characteristics among the study subjects

Variable	Total	Behavioral risk Factors for cardiovascular diseases (Yes, %)			
		Use of tobacco	Use of alcohol	Insufficient fruit & Veg.	physical inactivity
	*N* (%)	Yes, *n* (%)	Yes, *n* (%)	Yes, *n* (%)	Yes, *n* (%)
	426 (100)	91(21.36)	57 (13.38)	370 (86.9)	230 (53.99)
Age (years)		*p* = 0.006	*p* < 0.0001	*p* = 0.686	*p* = 0.004
< 20	102 (23.9)	33 (32.4)	22 (21.6)	86 (84.3)	65 (63.7)
20–34	290 (68.1)	45 (17.2)	21 (8.0)	254 (87.5)	143 (46.2)
35–45	34 (8.0)	13 (21.0)	14 (22.6)	30 (88.2)	22 (64.7)
Caste/ethnicity		*p* < 0.0001	*p* < 0.0001	*p* = 0.044	*p* < 0.0001
Dalit	71 (16.7)	31 (43.7)	30 (42.3)	68 (95.8)	17 (23.9)
Adibasi/ Janajati	91 (21.4)	27 (29.7)	7 (7.7)	79 (86.8)	37 (40.7)
Upper caste group	264 (62.0)	33 (12.5)	20 (7.6)	223 (84.5)	176 (66.7)
Origin		*p* = 0.542	*p* = 0.01	*p* = 0.235	*p* = 0.466
Hill	120 (28.2)	34 (19.9)	31 (18.1)	100 (83.3)	96 (56.1)
Terai	306 (71.8)	57 (22.4)	26 (10.2)	270 (88.2)	134 (52.5)
Religion		*p* = 0.05	*p* = 0.086	*p* = 0.835	*p* = 0.146
Hindu	391 (91.8)	79 (20.2)	49 (12.5)	340 (87.0)	207 (52.9)
Muslim/Buddhist	35 (8.2)	12 (34.3)	8 (22.9)	30 (85.7)	23 (65.7)
Women education		*p* < 0.0001	*p* < 0.0001	*p* = 0.005	*p* < 0.0001
No education	108 (25.4)	49 (35.3)	42 (30.2)	99 (91.6)	50 (36.0)
Primary	149 (35.0)	23 (19.5)	3 (2.5)	136 (91.2)	76 (64.4)
Secondary	79 (18.5)	7 (8.9)	5 (6.3)	65 (82.2)	52 (65.8)
Higher	90 (21.1)	12 (13.3)	7 (7.8)	70 (77.7)	52 (57.8)
Women occupation		*p* = 0.545	*p* = 0.002	*p* = 0.971	*p* = 0.623
Non-agriculture	292 (68.5)	60 (20.5)	29 (9.9)	254 (87.0)	132 (45.2)
Agriculture	134 (31.5)	31 (23.1)	28 (20.9)	116 (86.6)	64 (47.8)
Wealth Index		*p* < 0.0001	*p* < 0.0001	*P* < 0.0001	*p* < 0.0001
Lower	124 (29.1)	48 (38.7)	38 (30.6)	119 (93.5)	43 (34.7)
Second	100 (23.5)	13 (13.0)	5 (5.0)	80 (83.0)	53 (53.0)
Middle	84 (19.7)	12 (14.3)	4 (4.8)	80 (95.2)	53 (63.1)
Fourth	61 (14.3)	6 (9.8)	4 6.6)	52 (85.2)	47 (77.0)
Highest	57 (13.4)	12 (21.1)	6 (10.5)	39 (63.1)	34 (59.6)
Parity		*p* = 0.540	*p* = 0.021	*p* = 0.334	*p* = 0.329
Primi	167 (39.20)	32 (19.2)	17 (10.2)	149 (89.2)	91 (54.5)
1	112 (26.29)	29 (25.9)	19 (17.0)	98 (87.5)	63 (56.2)
2	58 (13.62)	13 (22.4)	3 (5.2)	61 (87.9)	35 (60.3)
3+	89 (20.89)	17 (19.1)	18 (20.2)	82 (92.1)	41 (46.1)

Note: *p* < 0.05 for statistical significance

Chi-square test demonstrated that participants’ baseline characteristics-(age, caste/ethnicity, women education, and wealth index), (age, caste/ethnicity, birth origin, women education, women occupation, wealth index and parity), (caste/ethnicity, women education and wealth index), and (age, caste/ethnicity, women education, and wealth index) were respectively, strongly associated with tobacco use, alcohol use, insufficient fruit and vegetable intake, and insufficient physical activity (*p* < 0.05) (Table 2).

The results of multiple regression analyses for risk factors of CVD are presented in Table 3. Expectant mothers aged 20–34 (AOR 2.11; 95% CI (0.97–4.57)), 35–45 (AOR 2.31; 95% CI (0.83–6.41)), of Terai origin (AOR 1.18; 95% CI (0.67–2.08)), Muslim/Buddhist (AOR 1.33; 95% CI (0.55–3.20)), engaged in agriculture (AOR 2.89; 95% CI (1.3–6.15)), belonging to the second wealth index (AOR 2.73; 95% CI (1.12–6.64)) were more likely to consume tobacco than their counterparts. Pregnant women in upper caste group (AOR 0.50; 95% CI (0.25–0.99)), and with a primary (AOR 0.20; 95% CI (0.8–0.50)) or higher education (AOR 0.32; 95% CI (0.12–0.81)) were less likely to consume tobacco compared with Dalit caste group and no education, respectively. Women with parity > 3(AOR 4.80; 95% CI (1.15–19.95)) were more likely to consume alcohol than those with ≤ 3 parity. However, women from Terai origin (AOR 0.43; 95% CI (0.21–0.90) and having primary education (AOR 0.15; 95% CI (0.52–0.48) were associated with less alcohol consumption.

**Table 3:** Adjusted Odds Ratios (aOR) for behavioral risk factors of CVD among rural women

Variable	Behavioral risk Factors for cardiovascular diseases (aOR, 95% CI)			
	Use of tobacco	Use of Alcohol	Insufficient Fruits and Vegetables	Physical inactivity
Age (Years)				
< 20	1.00	1.00	1.00	1.00
20–34	2.11 (0.97–4.57)	1.15 (0.33–3.96)	1.19 (0.32–4.43)	4.84 (2.22–10.57)**
35–45	2.31 (0.83–6.41)	1.99 (0.73–5.41)	1.41 (0.43–4.64)	0.6 (0.2–1.8)
Caste/ethnicity				
Dalit	1.00	1.00	1.00	1.00
Adibasi/ Janajati	0.31 (0.14–0.67)	0.26 (0.10–0.64)	0.51 (0.12–2.18)	5.14 (2.29–11.53)**
Upper caste group	0.50 (0.25–0.99)**	1.67 (0.58–4.82)	0.42 (0.14–1.24)	3.19 (1.72–5.92)**
Origin				
Hill	1.00	1.00	1.00	1.00
Terai	1.18 (0.67–2.08)	0.43 (0.21–0.90)**	2.06 (1.07–3.9)**	0.84 (0.51–1.3)
Religion				
Hindu	1.00	1.00	1.00	1.00
Muslim/others	1.33 (0.55–3.20)	1.01 (0.36–2.80)	2.1 (0.44–10.33)	2.94 (1.14–7.57)**
Women Education				
No education	1.00	1.00	1.00	1.00
Primary	0.20 (0.8–0.50)**	0.15 (0.52–0.48)**	0.36 (0.18–0.74)**	3.59 (1.58–8.14)**
Secondary	1.17 (0.40–3.38)	1.82 (0.39–8.52)	0.28 (0.18–3.65)	0.45 (0.21–0.94)**
Higher	0.32 (0.12–0.81)**	0.59 (0.16–2.24)	0.14 (0.07–2.33)	0.72 (0.33–1.55)
Women Occupation				
Non-agriculture	1.00	1.00	1.00	1.00
Agriculture	2.89 (1.3–6.15)**	2.02 (0.82–4.95)	0.70 (0.24–2.05)	1.4 (0.7–3.0)
Wealth Index				
Lower	1.00	1.00	1.00	1.00
Second	2.73 (1.12–6.64)**	0.77 (0.23–2.55)	0.16 (0.04–0.68)**	0.90 (0.40–2.03)
Middle	1.61 (0.64–4.08)	2.22 (0.51–9.68)	0.43 (0.12–1.43)	0.40 (0.16–0.95)**
Fourth	2.13 (0.71–6.39)	1.68 (0.37–7.63)	0.13 (0.03–0.54)**	0.29 (0.14–0.74)**
Highest	0.75 (0.29–1.94)	1.41 (0.33–6.01)	0.54 (0.18–1.61)	1.19 (0.48–2.91)
Parity (years)				
Primi	1.00	1.00	1.00	1.00
One	1.05 (0.53–2.08)	1.82 (0.65–5.07)	2.88 (1.16–7.13)**	0.85 (0.46–1.55)
Two	0.75 (0.31–1.82)	1.46 (0.48–4.39)	1.86 (0.60–5.73)	0.66 (0.30–1.45)
Three and above	1.13 (0.49–2.59)	4.80 (1.15–9.95)**	1.40 (0.46–4.28)	1.26 (0.63–2.55)

**p value < 0.05 (Significant)

Women from Terai origin (AOR 2.06; 95% CI (1.07–3.9)), primigravida (AOR 2.88; 95% CI (1.16–7.13)), primary education (AOR 0.15; 95% CI (0.52–0.48)), second wealth index (AOR 0.16; 95% CI (0.04–0.68)) and fourth wealth index (AOR 0.13; 95% CI (0.03–0.54)) were less likely to consume fruit and vegetable.

Regarding physical inactivity, respondents aged 20–34 years (AOR 4.84; 95% CI (2.22–10.57)), Adibashi/Janajati women (AOR 5.14; 95% CI (2.29–11.53)), upper caste (AOR 3.19; 95% CI (1.72–5.92)), Muslim/Buddhist (AOR 2.94; 95% CI (1.14–7.57)), primary education (AOR 3.59; 95% CI (1.58–8.14)), secondary education (AOR 0.45; 95% CI (0.21–0.94)), wealth indices middle (AOR 0.40; 95% CI (0.16–0.95)) and fourth (AOR 0.29; 95% CI (0.14–0.74)) were less likely to be physically inactive.

The results of multiple regression analyses for behavioral risk factors of CVDs among pregnant women with associated risk factors are presented in Table 4. Expectant mothers with primary level education (AOR 2.78; 95% CI (1.35–5.72)) were more likely to have ≥ 2 behavioral risk factors than illiterate women or women with a higher educational level.

**Table 4:** Prevalence and Adjusted Odds Ratios (aORs) of ≥ 2 behavioral risk factors for CVD among pregnant rural women

Variable	≥ 2 Behavioral risk Factors for cardiovascular diseases (aORs, 95% CI)		
	*N* (%)	aORs	95%CI
Age (Years)			
< 20	77 (75.5)	1.00	–
20–34	160 (61.1)	0.27	0.13–0.56**
35–49	46 (74.2)	0.91	0.37–2.24
Caste/ethnicity			
Dalit	49 (69.0)	1.00	–
Adibasi/Janajati	60 (65.9)	0.84	0.40–1.77
Upper caste group	174 (65.9)	0.94	0.45–1.97
Place of Origin			
Hill	115 (67.3)	1.00	–
Terai	168 (65.9)	0.89	0.55–1.44
Religion			
Hindu	256 (65.5)	1.00	–
Muslim/others	27 (77.1)	1.87	0.76–4.58
Women Education			
No education	88 (63.3)	1.00	–
Primary	89 (75.4)	2.78	1.35–5.72**
Secondary	50 (63.3)	1.28	0.63–2.61
Higher	56 (62.2)	1.15	0.56–2.35
Women Occupation			
Non-agriculture	197 (67.5)	1.00	–
Agriculture	86 (64.2)	0.59	0.31–1.11
Wealth Index			
Lower	87 (70.2)	1.00	–
Second	64 (64.0)	0.59	0.27–1.2
Middle	59 (70.2)	1.22	0.55–2.73
Fourth	42 (68.9)	0.98	0.41–2.30
Highest	31 (54.4)	0.36	0.16–0.84**
Parity (years)			
Primi	112 (67.1)	1.00	–
1	75 (67.0)	0.74	0.42–1.32
2	41 (70.7)	1.20	0.58–2.49
3+	55 (61.8)	0.77	0.40–1.47
Total	283 (100)	–	–

**p value < 0.05 (Significant)

Women aged 20–34 years (AOR 0.27; 95% CI (0.13–0.56)) had a lower chance for more than one behavioral risk factors than those of either lower or higher age group. Similarly, women belonging to the highest wealth index (AOR 0.36; 95% CI (0.16–0.84)) were less likely to have more than one behavioral risk factors compared to lower wealth quintile.

## Discussion

This study provides the prevalence and socio-economic predictors of the four major behavioral risk factors of CVDs: insufficient fruit and vegetable consumption, insufficient physical activity, use of tobacco and harmful alcohol consumption by pregnant women. We noted this as the first ever study from rural Nepal that focused on predicting behavioral risk factors of CVDs among pregnant women.

The most common behavioral risk factor of CVD in pregnant women is the insufficient intake of fruits and vegetables, which concurs with the findings from a nationwide survey on risk factors of non-communicable diseases and with other studies conducted in different parts of Nepal [6, 14, 30]. These studies reported that insufficient fruits and vegetables consumption among pregnant women could be attributed to poverty that might have caused rural women not to afford adequate amount of fruits and vegetables. The other studies also supported that low income level prevents from consuming adequate fruits and vegetables [31, 32]. Another possible reason for less intake of fruits and vegetables during pregnancy could be associated with seasonal and geographical availability of such foods. In Nepalese culture, fruits consumption is not considered a priority compared to main cereals in their daily meals, most importantly rice and wheat. Cultural practices to avoid more hot foods during pregnancy is prevalent due to the belief that pregnancy is considered as hot state [33]. A study conducted by Christian et al. [34] described that foods such as peppers, lime and tamarind, sweet foods and green leafy vegetables that are fibrous and hard to digest, have been avoided during pregnancy in Terai, Nepal. Likewise, it is believed that eating sufficient mangoes is considered as a cause of abortion. Some of the cultural beliefs for not eating sufficient foods include ‘causing harm to mother and/or baby’ and ‘not enough space for baby in stomach’ [34].

Lack of physical activity during pregnancy was the second most common behavioral risk factor of CVDs among our study subjects with a prevalence of 53.9%, which is similar to that found in other Nepalese studies [6, 35], but significantly greater than those found in nationwide non-communicable disease survey [36]. This could result from the general belief among Nepalese and promoted by some grassroot level health care providers that physical activity leads to miscarriage, poor fetal growth or premature delivery. A systematic review on physical activity during pregnancy suggests that some light-to-moderate physical activity is protective for maternal and child health outcomes such as pre-eclampsia, gestational hypertension and premature birth [37]. Physical activity guidelines around the world has recommended moderate intensity exercise such as brisk walking and other leisure time activities for pregnant women [38], therefore, appropriate counseling regarding optimal physical activity during pregnancy could reduce the risk of CVDs associated with inadequate physical activity.

High rates of tobacco use (21.3%) and alcohol consumption (13.3%) among pregnant women are other key findings from the present study. Previous studies from Nepal also identified a higher prevalence of tobacco and alcohol use during pregnancy [17, 39]. The reason behind increasing tobacco use could be the widespread availability of a range of tobacco products, which made it easier to access for everyone even in rural areas. One of the most common form of tobacco used in rural parts of south Asian countries, including Nepal is Bidi (a dried and crushed tobacco flakes rolled by hand in *tendu* (*Diospyrus*) leaf) that contains higher amount of nicotine and tar than cigarette [40].

Similarly, brewing different types of traditional alcoholic beverages at home in rural Nepal [41], most commonly by women, could be one of the common reasons of a high rate of alcohol consumption by pregnant women. In Nepal, a nationwide study reported that cereals (rice, barley and millet) and sugar are commonly used ingredients to prepare home-brewed alcohol. Ethanol concentration on home-brewed alcohol were 14.0% (3 to 40%) for distilled and 5.2% (1 to 18.9%) for non-distilled forms [41]. Locally brewed alcoholic beverages are considered culturally acceptable to drink even by pregnant women in some ethnic groups. This could be due to lack of awareness about the harmful effects of these products to mother and fetus during pregnancy. Health promotion strategies should, therefore, include general awareness programme to reduce these maternal behavior risk factors of CVDs.

Interestingly, our study demonstrated the association between educational level, age and wealth index of respondents with more than one behavioral risk factors of CVDs. Adjusting for potential confounders yielded more direct evidence of the contribution of these parameters to the prevalence of risk factors for CVDs among pregnant women in rural Nepal. Poor level of awareness on negative effects of behavioral risk factors of CVDs during pregnancy could be associated with low level of literacy. Similarly, poor access to adequate fruits and vegetables could be associated with poverty in rural areas. Several studies [39, 42, 43] are in line with our study findings. For example, level of education and age factors were associated with physical inactivity [42, 43]. Targeted educational campaigns and poverty reduction strategies should be recommended to reduce behavioral risk factors of CVDs among women in rural settings.

Despite our efforts to explore this important topic in the Nepalese context, this study should be evaluated in the light of some limitations. First, the information we collected was obtained by self-reporting, and thus, is subject to informant bias. Second, our study did not consider information about nutritional status of pregnant women (e.g. under or over nutrition status by body mass index) which is considered to be one of the major factors of understanding these risk factors [44]. Third, because the study was performed in southern plain area, its findings may not be generalizable for hilly or mountainous regions. Thus, it would be imperative to conduct similar types of studies in hilly and mountainous regions of Nepal, taking into consideration of the limitations of this study. Further, intervention studies to reduce prevalence of behavioral risk factors of CVDs during pregnancy and to improve birth outcome is recommended for Nepal.

## Conclusion

This study identifies a high prevalence of fruits and vegetables insufficiency, insufficient physical activity, tobacco use and alcohol consumption as behavioral risk factors of CVDs among pregnant women residing in rural Nepal and highlights the predictors of these behavioral risk factors. Health promotion strategies such as community-based awareness activities on how to reduce behavioral risk factors of CVDs and appropriate counseling regarding cardiovascular risk reduction ought to be routinely performed in antenatal clinics in rural Nepal. Furthermore, interventional studies should be considered to reduce risky behaviors in pregnancy associated with CVDs to improve maternal and child health outcomes.

## Abbreviations

AOR: Adjusted Odds Ratio
CI: Confidence Interval
CVDs: Cardiovascular Diseases
FCHVs: Female Community Health Volunteers
LMICs: Low- and Middle-income Countries
MCH: Maternal and Child Health
VDCs: Village Development Committees
WHO: World Health Organization

## References

[1] American Heart Association. What is cardiovascular disease? Available from: http://www.heart.org/en/health-topics/consumer-healthcare/what-is-cardiovascular-disease. Accessed 18 Dec 2017.

[2] World Health Organization. Cardiovascular diseases (CVD) fact sheet. Available from: http://www.who.int/en/news-room/fact-sheets/detail/cardiovascular-diseases-(cvds). Accessed 18 Dec 2017.

[3] Mendis S, Puska P, Norrving B. Global atlas on cardiovascular disease prevention and control. Geneva: World Health Organization; 2011.

[4] Vaidya A, Pokharel PK, Nagesh S, Karki P, Kumar S, Majhi S (2009). Prevalence of coronary heart disease in the urban adult males of eastern Nepal: a population-based analytical cross-sectional study. Indian Heart J.

[5] Maskey A, Sayami A, Pandey M (2003). Coronary artery disease: an emerging epidemic in Nepal. J Nepal Med Assoc.

[6] Dhungana RR, Devkota S, Khanal MK, Gurung Y, Giri RK, Parajuli RK, Adhikari A, Joshi S, Hada B, Shayami A (2014). Prevalence of cardiovascular health risk behaviors in a remote rural community of Sindhuli district, Nepal. BMC Cardiovasc Disord.

[7] World Health Organization. Global health risks: mortality and burden of disease attributable to selected major risks. Geneva: World Health Organization; 2009.

[8] Faden VB, Graubard BI, Dufour M (1997). The relationship of drinking and birth outcome in a US national sample of expectant mothers. Paediatr Perinat Epidemiol.

[9] Bailey BA, Sokol RJ (2011). Prenatal alcohol exposure and miscarriage, stillbirth, preterm delivery, and sudden infant death syndrome. Alcohol Res Health.

[10] Grewal J, Carmichael SL, Ma C, Lammer EJ, Shaw GM (2008). Maternal periconceptional smoking and alcohol consumption and risk for select congenital anomalies. Birth Defects Res A Clin Mol Teratol.

[11] Hackshaw A, Rodeck C, Boniface S. Maternal smoking in pregnancy and birth defects: a systematic review based on 173 687 malformed cases and 11.7 million controls. Hum Reprod Update. 2011. 10.1093/humupd/dmr022. 10.1093/humupd/dmr022 PMC3156888 21747128

[12] Gelson E, Gatzoulis MA, Steer P, Johnson MR (2009). Heart disease—why is maternal mortality increasing?. BJOG.

[13] Hall ME, George EM, Granger JP (2011). The heart during pregnancy. Rev Esp Cardiol.

[14] Aryal K, Neupane S, Mehata S, Vaidya A, Singh S, Paulin F, Madanlal R, Riley L, Cowan M, Guthold R (2014). Non communicable diseases risk factors: STEPS Survey Nepal 2013.

[15] Khaw KT, Wareham N, Bingham S, Welch A, Luben R, Day N (2008). Combined impact of health behaviours and mortality in men and women: the EPIC-Norfolk prospective population study. PLoS Med.

[16] Martin-Diener E, Meyer J, Braun J, Tarnutzer S, Faeh D, Rohrmann S, Martin BW (2014). The combined effect on survival of four main behavioural risk factors for non-communicable diseases. Prev Med.

[17] Niraul S, Jha N, Shyangwa P (2014). Alcohol consumption among women in a district of Eastern Nepal. Health Renaiss.

[18] Niraula SR, Shyangwa P, Jha N, Paudel R, Pokharel P. Alcohol use among women in a town of eastern Nepal. J Nepal Med Assoc. 2004;43(155):244–49.

[19] Singh JK, Kadel R, Acharya D, Lombard D, Khanal S, Singh SP (2018). ‘MATRI-SUMAN’ a capacity building and text messaging intervention to enhance maternal and child health service utilization among pregnant women from rural Nepal: study protocol for a cluster randomised controlled trial. BMC Health Serv Res.

[20] District Development Committee Dhanusha (2012). District Profile of Dhanusha, District Development Committee.

[21] Central Bureau Statistics (2014). General and Social Characteristics Tables: Household and Population, Age-Sex Distribution, Relationship, Marital Status and Religion, National Population and Housing Census 2011.

[22] District Health Office Dhanusha. District health profile of Dhanusha, Nepal. Edited by Office MoHaPDH, Dhanusha; 2013.

[23] District Development Committee Dhanusha, Ministry of Local Development. A profile of Dhanusha District of Nepal. Edited by Ministry of Local Development, Janakpur and District Development Committee, Dhanusha; 2012.

[24] Karki K, Dahal B, Regmi A, Poudel A, Gurung Y (2008). WHO STEPS Surveillance: Non Communicable Diseases Risk Factors Survey. 2008.

[25] Agudo A, Joint F (2005). Measuring intake of fruit and vegetables [electronic resource].

[26] Organization WH. Obesity: preventing and managing the global epidemic. Geneva: World Health Organization; 2000. 11234459

[27] Ministry of Health and Population NE, ICF International. Nepal Demographic and Health Survey 2011. Kathmandu: Nepal Ministry of Health and Population, New ERA and Maryland: ICF International Calverton; 2012.

[28] Acharya D, Khanal V, Singh JK, Adhikari M, Gautam S (2015). Impact of mass media on the utilization of antenatal care services among women of rural community in Nepal. BMC Res Notes.

[29] Barbosa Filho VC, de Campos W, Bozza R, da Silva LA (2012). The prevalence and correlates of behavioral risk factors for cardiovascular health among Southern Brazil adolescents: a cross-sectional study. BMC Pediatr.

[30] Oli N, Vaidya A, Thapa G. Behavioural risk factors of noncommunicable diseases among Nepalese urban poor: a descriptive study from a slum area of Kathmandu. Epidemiol Res Int. 2013;2013:13.

[31] Hall JN, Moore S, Harper SB, Lynch JW (2009). Global variability in fruit and vegetable consumption. Am J Prev Med.

[32] Valmórbida JL, Vitolo MR (2014). Factors associated with low consumption of fruits and vegetables by preschoolers of low socio-economic level. J Pediatr.

[33] Christian P, West K, Katz J, Kimbrough-Pradhan E, LeClerq S, Khatry S, Shrestha S (2004). Cigarette smoking during pregnancy in rural Nepal. Risk factors and effects of β-carotene and vitamin A supplementation. Eur J Clin Nutr.

[34] Christian P, Bunjun Srihari S, Thorne-Lyman A, Khatry SK, LeClerq SC, Shrestha SR (2006). Eating down in pregnancy: exploring food-related beliefs and practices of pregnancy in rural Nepal. Ecol Food Nutr.

[35] Bhandari B, Bhattarai M, Bhandari M, Ghimire A, Pokharel PK (2014). Prevalence of other associated risk factors of Cardiovascular Disease among Hypertensive patients in Eastern Nepal. Nepal Heart J.

[36] Aryal K, Thapa P, Mehata S, Vaidya A, Pandey A, Bista B, Pandit A, Dhakal P, Karki K, Dhimal M. Alcohol Use by Nepalese Women: Evidence from Non Communicable Disease Risk Factors STEPS Survey Nepal 2013. J Nepal Health Res Counc. 2015;13(29):1–6. 26411705

[37] Schlüssel MM, Souza EB, Reichenheim ME, Kac G (2008). Physical activity during pregnancy and maternal-child health outcomes: a systematic literature review. Cad Saude Publica.

[38] Evenson KR, Barakat R, Brown WJ, Dargent-Molina P, Haruna M, Mikkelsen EM, Mottola MF, Owe KM, Rousham EK, Yeo S (2014). Guidelines for physical activity during pregnancy: comparisons from around the world. Am J Lifestyle Med.

[39] Chasan-Taber L, Schmidt MD, Pekow P, Sternfeld B, Manson J, Markenson G (2007). Correlates of physical activity in pregnancy among Latina women. Matern Child Health J.

[40] Rahman M, Fukui T (2000). Bidi smoking and health. Public Health.

[41] Thapa N, Aryal KK, Paudel M, Puri R, Thapa P, Shrestha S, Shrestha S, Stray-Pedersen B (2015). Nepalese homebrewed alcoholic beverages: types, ingredients, and ethanol concentration from a nation wide survey. J Nepal Health Res Counc.

[42] Dumith SC, Domingues MR, Mendoza-Sassi RA, Cesar JA (2012). Physical activity during pregnancy and its association with maternal and child health indicators. Rev Saude Publica.

[43] Nascimento SL, Surita FG, Godoy AC, Kasawara KT, Morais SS (2015). Physical activity patterns and factors related to exercise during pregnancy: a cross sectional study. PLoS One.

[44] Hashmi AH, Paw MK, Nosten S, Darakamon MC, Gilder ME, Charunwatthana P, Carrara VI, Wickramasinghe K, Angkurawaranon C, Plugge E (2018). ‘Because the baby asks for it’: a mixed-methods study on local perceptions toward nutrition during pregnancy among marginalised migrant women along the Myanmar-Thailand border. Glob Health Action.

